# Programmed Effects in Neurobehavior and Antioxidative Physiology in Zebrafish Embryonically Exposed to Cadmium: Observations and Hypothesized Adverse Outcome Pathway Framework

**DOI:** 10.3390/ijms17111830

**Published:** 2016-11-02

**Authors:** Sander Ruiter, Josefine Sippel, Manon C. Bouwmeester, Tobias Lommelaars, Piet Beekhof, Hennie M. Hodemaekers, Frank Bakker, Evert-Jan van den Brandhof, Jeroen L. A. Pennings, Leo T. M. van der Ven

**Affiliations:** 1 Centre for Health Protection, National Institute for Public Health and the Environment (RIVM), Bilthoven 3720BA-1, The Netherlands; piet.beekhof@rivm.nl (P.B.); hennie.hodemaekers@rivm.nl (H.M.H.); frank.bakker@rivm.nl (F.B.); jeroen.pennings@rivm.nl (J.L.A.P.); 2 Centre for Environmental Quality, National Institute for Public Health and the Environment (RIVM), Bilthoven 3720BA-1, The Netherlands; evert-jan.van.den.brandhof@rivm.nl

**Keywords:** developmental origins of health and disease (DOHaD), programming, epigenetics, DNA methylation, cadmium, glutathione, *S*-adenosyl-methionine, oxidative stress, adverse outcome pathway (AOP), zebrafish

## Abstract

Non-communicable diseases (NCDs) are a major cause of premature mortality. Recent studies show that predispositions for NCDs may arise from early-life exposure to low concentrations of environmental contaminants. This developmental origins of health and disease (DOHaD) paradigm suggests that programming of an embryo can be disrupted, changing the homeostatic set point of biological functions. Epigenetic alterations are a possible underlying mechanism. Here, we investigated the DOHaD paradigm by exposing zebrafish to subtoxic concentrations of the ubiquitous contaminant cadmium during embryogenesis, followed by growth under normal conditions. Prolonged behavioral responses to physical stress and altered antioxidative physiology were observed approximately ten weeks after termination of embryonal exposure, at concentrations that were 50–3200-fold below the direct embryotoxic concentration, and interpreted as altered developmental programming. Literature was explored for possible mechanistic pathways that link embryonic subtoxic cadmium to the observed apical phenotypes, more specifically, the probability of molecular mechanisms induced by cadmium exposure leading to altered DNA methylation and subsequently to the observed apical phenotypes. This was done using the adverse outcome pathway model framework, and assessing key event relationship plausibility by tailored Bradford-Hill analysis. Thus, cadmium interaction with thiols appeared to be the major contributor to late-life effects. Cadmium-thiol interactions may lead to depletion of the methyl donor *S*-adenosyl-methionine, resulting in methylome alterations, and may, additionally, result in oxidative stress, which may lead to DNA oxidation, and subsequently altered DNA methyltransferase activity. In this way, DNA methylation may be affected at a critical developmental stage, causing the observed apical phenotypes.

## 1. Introduction

Non-communicable diseases (NCDs) are characterized by long duration and slow progression, resulting in extensive patient suffering and treatment, and are associated with huge medical costs; the World Economic Forum predicted accumulation of NCD related costs of up to $47 trillion worldwide over the period 2011–2030 [1]. Currently, NCD prevention campaigns focus on four adult life-style risk factors: physical inactivity, the harmful consumption of alcohol, unhealthy diets and tobacco use. Recent studies, however, suggest that sensitivity to develop NCDs may arise from perinatal conditions, including exposure to environmental chemicals and nutritional imbalances [2]. This paradigm is known as the developmental origins of health and disease (DOHaD) concept. Normal homeostasis in the organism is based on the programming of cell functions during embryogenesis and further early life stages. This dynamical and flexible process allows adaptations of the organism to its future environment. Altered programming, due to an insult at a critical, sensitive period of early life, leads to changed cell or tissue function, and subsequently to permanent effects on structure, physiology and metabolism (homeostasis) [3].

Epigenetics is a suggested mechanism of early-life programming. During embryonic life stages, cell-specific epigenetic profiles are defined, which control cellular functions and thereby cell differentiation. An alteration of the epigenetic make-up can lead to changes in gene expression, and thus in cell and tissue functions. Epigenetic regulation is established at several levels, of which the most studied are DNA methylation, histone modifications and microRNAs. DNA methylation, which comprises the covalent transfer of a methyl group from the methyl donor *S*-adenosyl-methionine (SAM) to the 5′ position of the cytosine pyrimidine ring by a DNA methyltransferase (DNMT), is a key regulator of cell differentiation. Most methylated cytosines are found adjacent to a 3′ guanine (CpG) and are mostly present in CpG rich areas called CpG islands. Hypermethylation of CpG islands in promoter regions is associated with silenced genes and hypomethylation with active genes [4]. During embryogenesis, a new DNA methylation landscape is created by de novo DNMTs 3A and 3B. This developmental plasticity could be susceptible to interference, which is thus proposed as a key mechanism for the disruption of programming [2].

Support for a link between chemical exposure early in life and late onset disease comes from both epidemiological and experimental studies, but the evidence is anecdotal and mechanistic support, even through differential DNA methylation, is limited. Recent reviews summarize experimental approaches and models that could be employed to investigate mechanisms of DOHaD and associated predictive biomarkers [5,6]. One such suggested model is *Danio rerio* (zebrafish), and therefore we previously explored effects on DNA methylation after embryonal exposure in this model with environmental contaminants [7]. Here, we expand that study with investigations into delayed effects of embryonal exposure to one such contaminant, cadmium. Cadmium has known epigenetic effects [7,8] and is a ubiquitous environmental pollutant leading to chronic low-dose exposure in humans through vegetables, cereals and tobacco [9]. In the experimental part of this study, zebrafish embryos were exposed (0–72 h post fertilization, hpf) to cadmium (CdCl_2_). After the early-life exposure, growth was continued without further exposure. At approximately 10 weeks of age, juvenile zebrafish were analyzed for apical phenotypes. Antioxidative parameters were chosen as an endpoint because oxidative stress is an important underlying factor in many chronic diseases which are characterized by chronic low grade inflammation, including chronic metabolic disease and autoimmune disease [10,11]. Neurobehavior was selected as an endpoint because delayed onset learning disabilities and behavior problems have been suggested as an area of programmed effects after chemical exposure early in life [12].

Although we observed both DNA methylation effects in the embryo and a changed adult phenotype, the mechanism of this association is not obvious. In the second part of this study, we therefore searched the literature for relevant data which could support and substantiate this link, and subsequently identify parameters that can be used to predict the outcome of the embryonal exposure in the adult animal. For these purposes, we structured the retrieved data using the adverse outcome pathway (AOP) model framework. AOPs sequentially describe the events from the first interaction of the stressor with the biological system (the molecular initiating event, MIE), via further events at increasingly high levels of biological complexity (key events, KE) to an apical phenotype, i.e., an adverse health effect [13]. Each step in an AOP is linked to the next by a key event relationship (KER). Although AOPs are not chemical-specific and describe generalized motifs of biological responses to an MIE, leading to an AO through one or multiple KEs, the programmed effects induced by embryonic cadmium exposure may provide an AOP case study [14].

## 2. Results

### 2.1. Embryotoxicity

Embryotoxicity of CdCl_2_ at 72 hpf was observed with a critical effect dose at the 5% effect level (CED05) of 32.2–67 μM in duplicate experiments, with no hatching as the observed sublethal effect, probably due to delayed development. Embryo survival was markedly reduced at 100 μM (40% compared to 80%–90% in lower concentrations).

### 2.2. DNA Methylation

**Figure 1:**
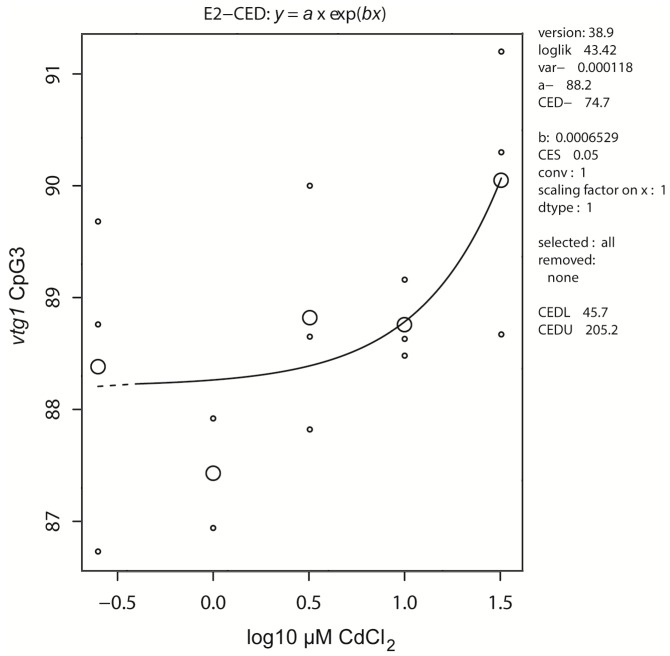
Dose response of methylation in *vtg1* CpG3 in whole embryo extracts after exposure to cadmium. Each small dot represents an individual sample consisting of 20 pooled embryos. Large circles are median values per concentration (controls, *n* = 6; exposed, *n* = 3). The right-hand legend is the standard output of the PROAST software, showing the software version; loglik (log likelihood) as a statistical measure of the selected model, var (variance) as a statistical descriptor of the dataset, a–d as parameters that describe the model (a, background; b, sensitivity; c, maximal effect; d, steepness; c and d appear in more complex models); CED, critical effect dose, calculated at a selected critical effect size (CES), and with the lower (CEDL) and upper (CEDU) bound of the 95% confidence interval. The other parameters show possible adaptions to the analysis or output, i.e., data transformation, selection, removal, and scaling.

The previously reported effects in the *vasa* promoter were not reproduced in a repeated experiment, where effects were observed in *cyp19a2* and *vtg1* CpGs (Table 1), the latter illustrated in Figure 1. All observed effects were at embryotoxic concentrations and may, altogether, be non-specific bystander effects.

### 2.3. DNA Oxidation

**Figure 2:**
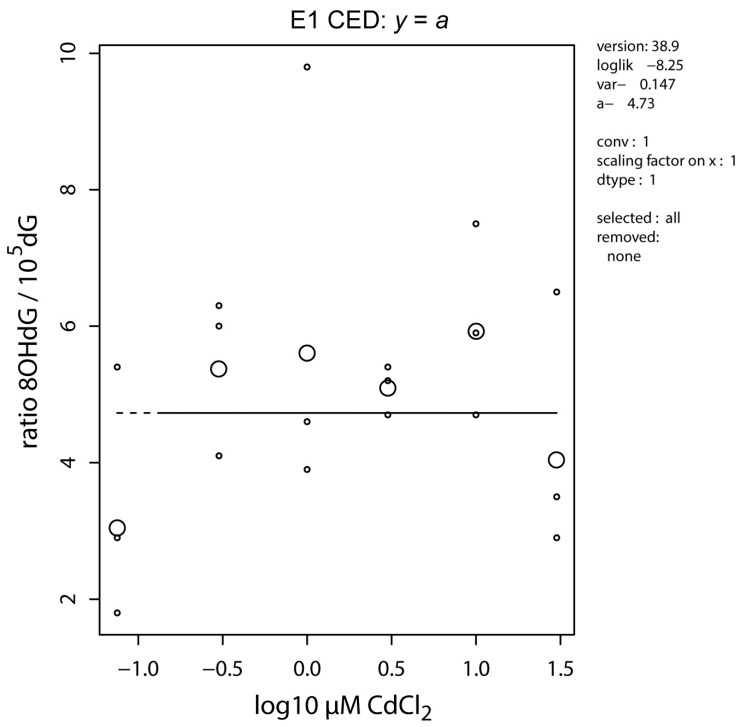
Dose response of the ratio of 8-OHdG/10^5^dG as a measure of DNA oxidation. Small dots are individual observations (pools of 40 embryos), large circles represent median values per concentration (*n* = 3).

The ratio of 8-OHdG/10^5^dG in whole embryo extracts did not show a significant dose-response effect when analyzed over the entire concentration range (Figure 2). However, a statistically significant concentration-dependent increase was observed after reanalysis of the data without the toxic top concentration (see above), suggesting that other toxic events could have interfered with the oxidizing effect of cadmium on the DNA at the higher concentration of 32.2 µM.

### 2.4. Neurobehavioral Studies

**Figure 3:**
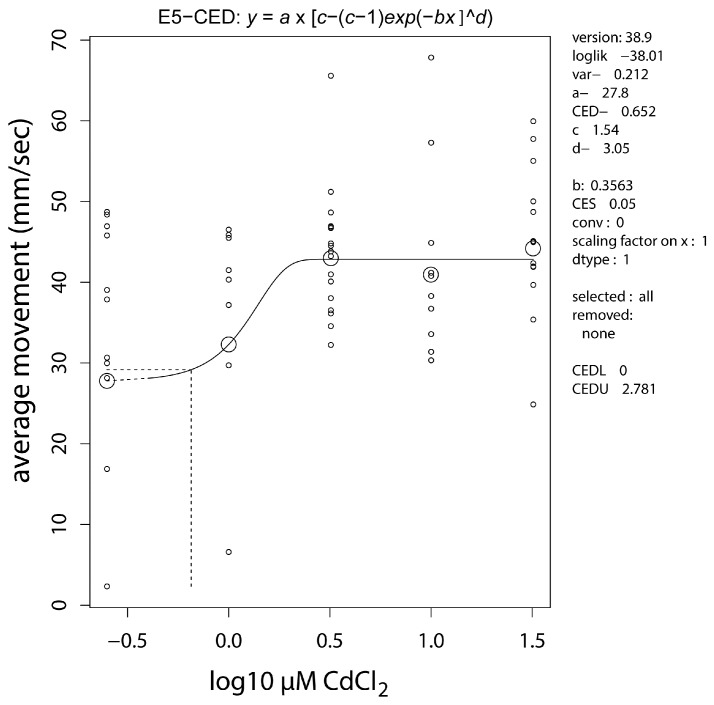
Dose-response analysis of average movement of embryonically cadmium exposed, adult zebrafish. The modelled response shows an increase of 54% at the plateau (c-parameter). A 5% increase (CES = 0.05) is calculated at the critical effect dose (CED) of 0.65 µM CdCl_2_. Small circles, individual measurements; large circles, median values per concentration.

Baseline movement of the zebrafish during a ten minute period showed a positive dose response, suggesting hyperactivity induced by embryonic cadmium exposure (Figure 3).

**Figure 4:**
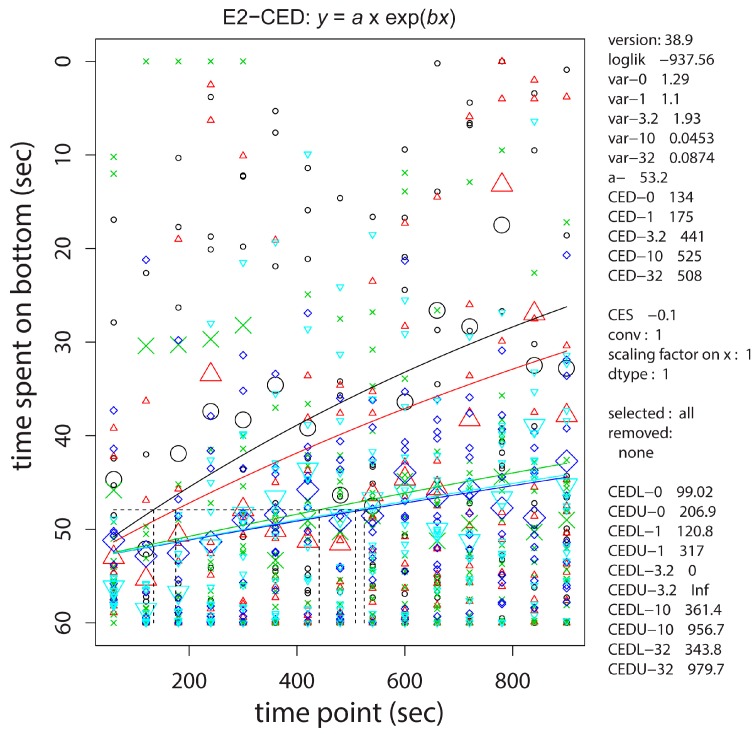
Bottom dwelling response of embryonically cadmium exposed adult zebrafish after transfer to a novel environment. Each column of data points represents time spent in the bottom third of the tank in the preceding 60 s. Small and large circles are individual and median values, respectively. The embryonic exposure groups are statistically significantly separated and are shown in black, red, green, dark blue, light blue, representing 0, 1, 3.2, 10, 32 μM of embryonic CdCl_2_ exposure, respectively.

In the novel tank diving test, time-response analysis with PROAST, using concentration as a covariate, showed that the time spent at the bottom of the tank (the stress response) persisted longer with an increasing concentration of the embryonic cadmium exposure (Figure 4). This analysis separates the control and lowest dose from the intermediate dose, then the two highest doses. This separation can also be read from the time it takes until one tenth of the response faded, given as CEDs 134, 175, 441, 525, 508 s for the 0, 1, 3.2, 10, and 32 µM groups respectively (right hand legend of Figure 4), as a measure for the observed statistically significant different time response between various concentrations. Associated with these measures are the lower and upper bounds of the confidence interval of the CED (the CED-L and CED-U), which have a ratio <5 for all concentration curves, except 3.2 μM, indicating acceptable small confidence intervals for the data set.

No statistically significant dose responses were observed in the tapping response test, nor in the background preference tests (dark-light, color; not shown).

### 2.5. Antioxidative Parameters

**Figure 5:**
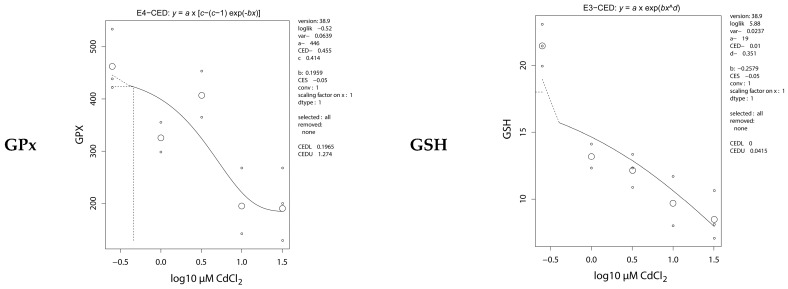

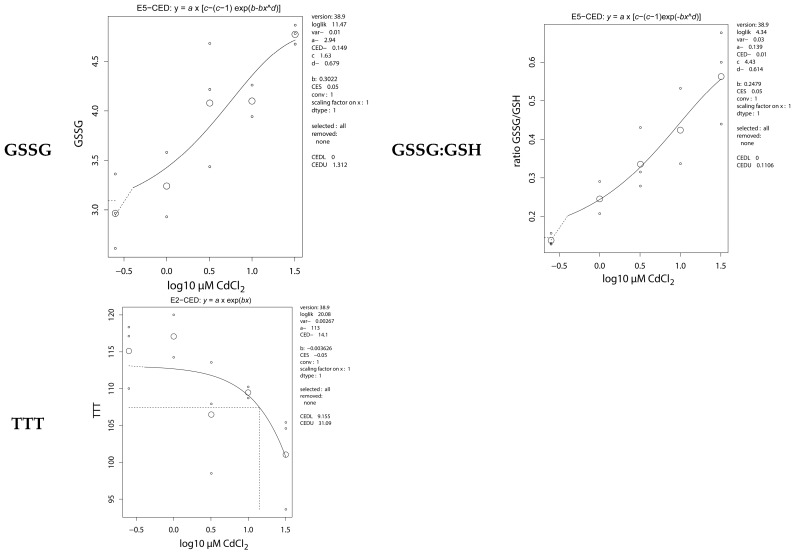
Dose-response analysis of background antioxidative parameters in the carcass of adult zebrafish exposed to cadmium during early embryonic life (0–72 hpf).

In the juvenile zebrafish exposed to cadmium as embryos, dose responses were observed for the antioxidative parameters glutathione peroxidase (GPx, decrease), reduced glutathione (GSH, decrease), oxidized glutathione (GSSG, increase), GSSG:GSH ratio (increase), and total thiols (TTT, decrease). Results were similar in the carcass (Figure 5) and organ extracts (not shown). No effects were observed on activity of the enzymes glutathione reductase (GR) and superoxide dismutase (SOD), nor on biological antioxidant potential (BAP). The CEDs at the 5% effect level of the significant dose responses are summarized in Table 2. CEDs were extrapolated from the dose-response curves at a concentration below the lowest exposure concentration of 1 μM, except for TTT in the carcass.

**Figure 6:**
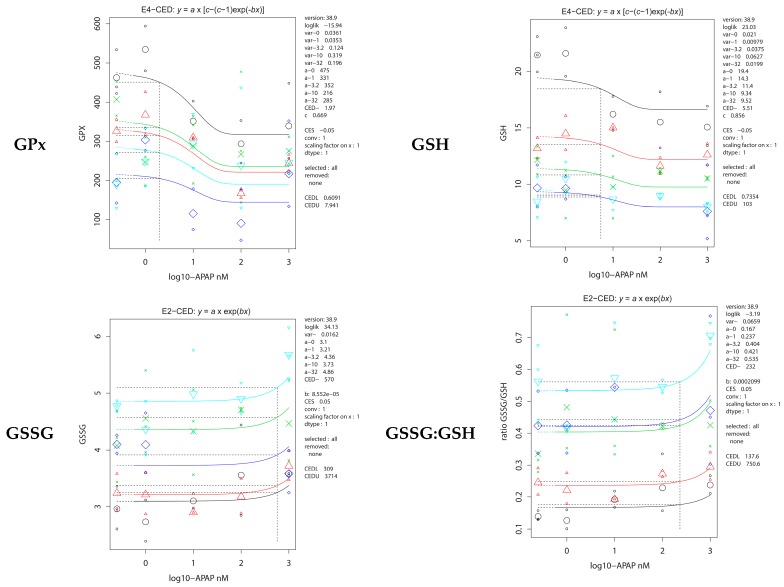

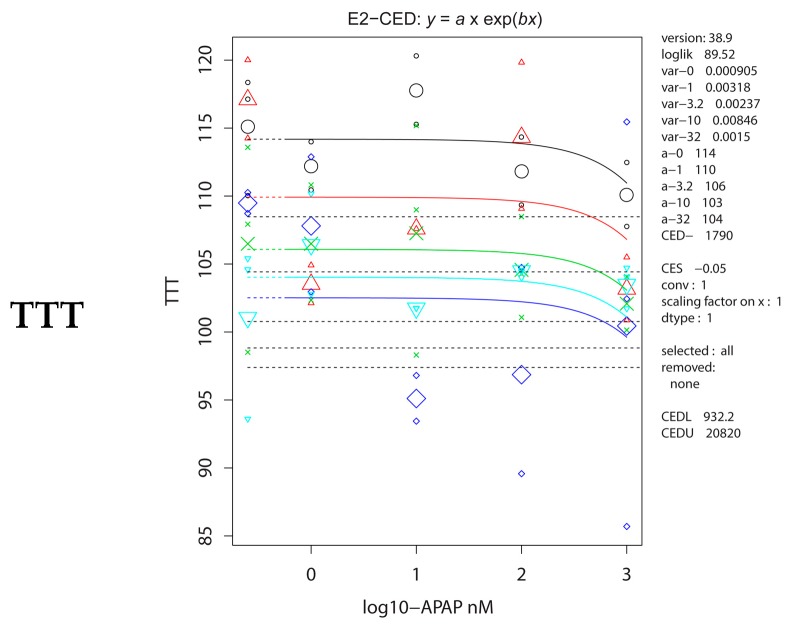
Dose response of antioxidative parameters in the carcass of adult zebrafish exposed to cadmium during early embryonic life (0–72 hpf) after a challenge with APAP. *X*-axis: APAP concentration; *Y*-axis: response; CdCl_2_ concentration groups are statistically significantly separated and shown as black—0 μM; red—1 μM; green—3.2 μM; dark blue—10 μM; light blue—32 μM CdCl_2_ at embryonic exposure.

A challenge with acetaminophen (APAP) induced an APAP dose dependent decrease of GPx, GSH and TTT, and increases in GSSG and ratio GSSG:GSH. For all five parameters, these APAP-induced effects ran parallel for the control and cadmium exposed fish, which therefore did not respond differently (Figure 6); CEDs for the various cadmium concentrations were therefore the same within each parameter set.

## 3. Discussion and Hypothesized AOP Framework

This study was initiated to investigate long term effects of embryonic exposure to cadmium in zebrafish. In adult fish, we analyzed neurobehavior and antioxidative physiology functions, because these are endpoints related to chronic non-communicable disease showing increased incidences in humans [10,11,15]. In a recent study, with a similar setup as ours regarding exposure methods and cadmium concentrations, the effects of cadmium exposure on behavior, antioxidative parameters and immunotoxicity were investigated in 4-day-post-fertilization larval zebrafish [16]. That study showed that the effects that we measured in adult fish were already present directly after exposure, as indicated by a dose dependent decrease of GSH concentrations and GPx activity, and increased GPx mRNA expression, possibly to compensate for the decrease in GPx activity. A neurobehavioral effect was detected as an altered swimming distance in a dark cycle given during daytime. These results indicate that the cadmium-induced apical phenotypes observed in our study are persisting after being established during the early-life exposure.

Observed baseline locomotion was readily reproducible among individuals, allowing detection of a dose-dependent increase of activity at adult age after embryonic cadmium exposure. However, large interindividual variation was observed in other neurobehavioral endpoints, and large study populations are required to detect possible effects. Neurobehavior testing in zebrafish is an upcoming discipline, and although many tests have been described, a recent workshop concluded that standardization and harmonization of such testing is needed—Fish and amphibian embryos as alternative models in toxicology and teratology. On 1–2 December 2014, Aulnay-sous-Bois/Paris, France—perhaps along the lines of a recently published zebrafish behavior catalog [17]. Part of the standardization may be the ambient condition in which the fish are raised and maintained, e.g., accustomization to ambient noise which may affect the response to tapping of the tank. One exception is the commonly used novel tank stress response test [18], which despite of high variance within the population (see Figure 4) still performed well in our situation, showing that zebrafish recovered slower from the stress induced by transfer to a novel tank with an increasing concentration of embryonic cadmium. This hyperresponsiveness may be in line with the hyperactivity observed in the baseline locomotion. Others showed that embryonic cadmium also delayed zebrafish antipredatory responses in later life [19]. Altogether, altered neurobehavior after embryonic cadmium at subtoxic concentrations may be caused by altered neuronal signaling, such as that observed in rats where perinatal cadmium exposure has been related to effects on neurotransmitters including dopamine and 5-hydroxytryptamine [20].

Long-term effects in neurobehavior in zebrafish are not unique for cadmium, because these were also observed after embryonic exposures to 3,3′,4,4′,5-pentachlorobiphenyl (PCB 126) [21] or alcohol [22,23]. Apparently, embryonic exposure to chemicals can disrupt cognitive functions, or more specifically, behavioral mechanisms that allow for (quick) adaption to (stressful) situations, with obvious consequences for fitness in competition, reproduction, and survival in the fish, and, by extrapolation, also possible functional deficits in humans.

In antioxidative physiology analysis in adult fish, multiple parameters showed alterations; a dose–response relationship was observed between embryonic cadmium concentration and the (interdependent) GSH, GSSG, GPx and TTT levels. The cadmium-dependent decrease of GSH was accompanied by an increase of GSSG, leading to an increased ratio of GSSG to GSH. Although exposure of these adult fish was only during embryonic life (0–72 hpf), and the body mass of the fish increased by an estimated factor of 500 (0.4 and 200 mg at 72 hpf and 10 weeks, respectively), it cannot be excluded that some residual cadmium remained, affecting e.g., relative organ ratios, and thereby these oxidative stress parameters. Otherwise, these observations may arise from programming effects leading to an altered homeostatic setpoint of the GSH/GSSG metabolism, or, alternatively, to programmed effects in e.g., mitochondrial, peroxisomal or inflammatory functions, followed by an induction of a permanent state of oxidative stress [24]. Increased GSH depletion due to exhaustion of other ROS scavengers [25] seems unlikely, since no effects were observed for BAP and SOD. The permanent state of oxidative stress, as indicated by the observed altered antioxidative physiology parameters in the adult fish is, in humans, associated with chronic disease [10,11]. On the other hand, cadmium exposure during early embryonic life appeared to have no effect on counteracting APAP-induced oxidative stress.

Altogether, the experimental results showed that embryonic cadmium exposure induces late life effects in neurobehavior and antioxidative parameters in zebrafish, confirming that normal programming during embryogenesis can be disturbed by this environmental contaminant, in line with the DOHaD concept. In the next section, possible cadmium-specific pathways which may lead to the observed apical phenotypes altered neurobehavior and antioxidative physiology, are explored in a structured way, by defining key events (KEs) and their relations (KERs), to arrive at a conceptual adverse outcome pathway framework.

### 3.1. Neurobehavior

**Figure 7:**
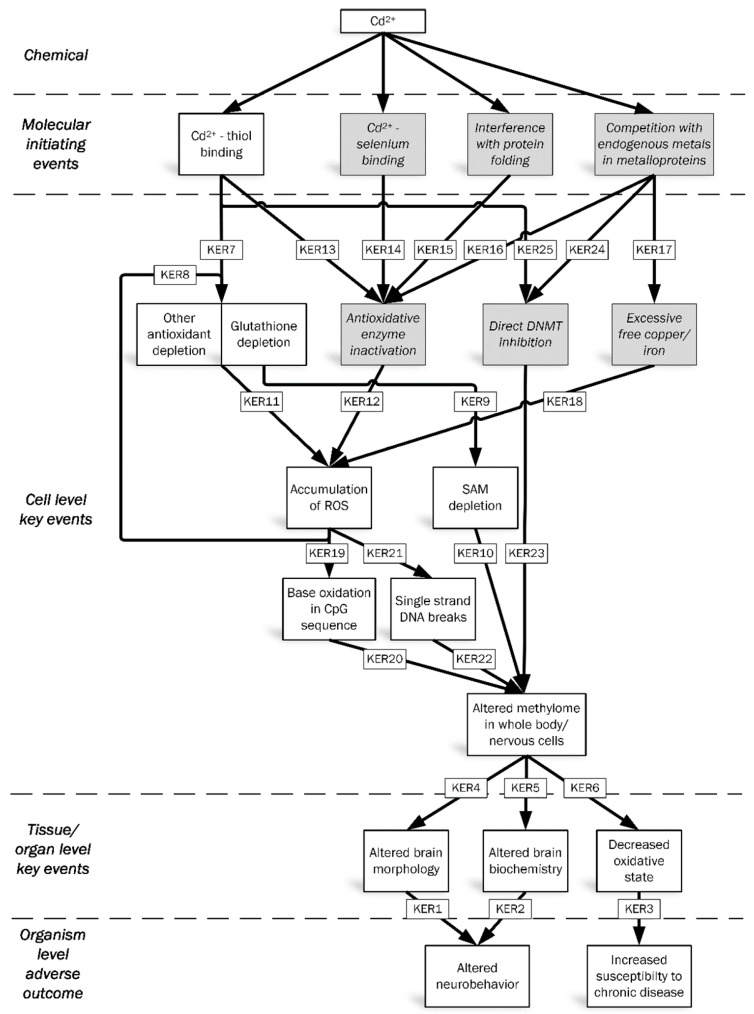
Hypothesized adverse outcome pathway (AOP) framework for embryonic subtoxic cadmium exposure leading to long term altered neurobehavior and antioxidative physiology. White and grey boxes, high and low probability key events, respectively. Numbering of KERs follows the order of description in the text.

The neurobehavioral experiments showed that embryonic, subtoxic cadmium leads to hyperactivity and impaired stress handling in adult zebrafish, indicating altered brain function. This altered brain function may be caused by decreased neuronal communication, which may have originated from changes in brain morphology and/or brain biochemistry (Figure 7, KERs 1 and 2), and the specific case of affected neurotransmitters after embryonic cadmium in rats was referred to above. In principle, such effects can be inflicted through two routes, i.e., developmental brain damage which persists through life, or altered epigenetic programming during perinatal exposure. Direct cadmium-induced neurotoxic effects are known to occur, e.g., through oxidative damage and hormone interactions [26]. However, in our study, altered neurobehavior was already observed at 67-fold lower concentrations than the 5% critical effect concentration for observable embryotoxic effects. Although not fully excluding direct neurotoxicity, this difference provides empirical support for more subtle processes, i.e., epigenetic programming, to play a role, rather than direct developmental toxicity. Epigenetic programming is further supported by the observed effects in oxidative stress response (next paragraph), thus providing a common mechanism for effects in two unrelated functions.

### 3.2. Oxidative Stress

Oxidative stress, mediated by low-grade chronic inflammation, plays an important role in the pathogenesis of chronic diseases [10,11] (Figure 7, KER3). Our embryonically cadmium exposed zebrafish had altered antioxidative physiology at adult age. No other studies describing delayed effects in antioxidative physiology after early-life cadmium exposure were retrieved, although such effects were observed with other early-life stressors. For instance, prenatal exposure to cigarette smoke induced increased renal ROS and oxidative DNA damage in mice up to postnatal day 90 [27]; neonatal exposure to corticosterone in mice induced increased lipid oxidation and decreased levels of antioxidants at postnatal day 120 [28]; and renal stress markers superoxide and NADPH-oxidase were increased in 16 week old male rats after in utero growth restriction [29]. These observations show that antioxidative physiology is subject to programming effects, supporting a similar role of embryonic exposure to cadmium in zebrafish.

### 3.3. Epigenetics

Alteration of the epigenetic profile is a proposed underlying mechanism for embryonic programming [30], and could thus establish the basis for KERs 4–6 (Figure 7).

As mentioned above, our zebrafish embryos showed cadmium-induced alterations on DNA methylation, which is a first layer of epigenetic regulation, largely established during early development. Although site-specific, these effects were only observed at embryotoxic concentrations and were probably random because of inconsistency among replicate experiments. These observations do not directly support DNA methylation effects as a specific mechanism, and other, mechanistically related loci may need to be analyzed in a tissue-specific manner. However, other models supported specific DNA methylation changes related to cadmium exposure, as reviewed by Ray et al. [31]. One reviewed study reported global and specific promoter hypermethylation and associated gene silencing of four DNA repair genes (*MSH2*, *ERCC1*, *XRCC1*, *OGG1*) in cadmium transformed human bronchial cells, together with overexpression of *DNMT1* and *DNMT3A*. All effects could be restored by the methylation inhibitor 5-aza-2′-deoxycytidine (5-aza) [32]. A second study observed cadmium-induced promoter hypomethylation and increased expression of *11-β-HSD2* in human choriocarcinoma cells [33]. Increased *DNMT3B* expression was observed in cadmium transformed prostate epithelial cells, associated with promoter hypermethylation and decreased expression of the tumor suppressor genes *RASSF1A* and *p16*, which was restored by the addition of 5-aza [34]. Furthermore, four studies in the Ray et al. review reported either global hypermethylation or hypomethylation and both site-specific hyper- and hypomethylation has been reported in genome-wide studies. The opposite effects of cadmium on DNA methylation may be explained by cell, tissue, or model specific responses. Although cadmium has confirmed effects on DNA methylation, in some cases associated with an altered phenotype, such causal associations could not be retrieved for the specific phenotypes under study (KERs 4–6). On the other hand, such relations are known for other toxicants, such as iron-ascorbate in human epithelial colorectal adenocarcinoma cells, where induced DNA methylation effects were related to altered antioxidative physiology, which could be restored by 5-aza. [35]. Regarding neurobehavior, prenatal, low concentration exposure to bisphenol A affected *Dnmt1* and *Gad67* expression in rat basolateral amygdala, leading to long term anxiety-like behavior [36]. Although these observations were not induced by cadmium, they support a link between chemically induced altered DNA methylation, gene expression, and altered programming of the studied phenotypes.

Histone modifications, which is another layer of epigenetic regulation, has also been observed as a target of cadmium-induced effects, particularly in in vitro studies. Thus, cadmium exposure led to altered lysine methylation in the promoter region of metallothionein 3 (MT3), and histone modifications associated with reactivation of the gene from a silenced state [37]. In another study, cadmium blocked histone modifications by inhibition of histone modifying enzymes [38], but molecular mechanisms by which cadmium alters histone modifications are not always clear. Also, the consequences of cadmium induced histone modifications for programming have not yet been established. Altogether, there is insufficient support to consider histone modifications as a KE in our pathway.

Cadmium has also been shown to affect the expression of multiple miRNAs, a third layer of epigenetic regulation, in human hepatoblastoma cells [39], but the lack of evidence for an intermediate role of miRNAs in early life programming in general makes this an unlikely scenario after embryonic cadmium exposure.

Altogether, cadmium appears to have an effect on multiple levels of epigenetic regulation of cell functions, but cadmium-induced DNA methylation alteration is the only pathway that has been associated with a specific phenotype, supporting a causal role of an altered methylome in the tissue/organ level KEs under study (Figure 7, KERs 4–6). Direct cadmium-related data on intermediate KEs on transcriptomic, proteomic and metabolomic levels, which should be included in these KERs, are limited.

### 3.4. Mechanisms Leading to Methylome Alteration

Since, based on available data, cadmium-induced effects on DNA methylation appear to be a plausible mechanism underlying altered programming, the next step is to identify pathways through which cadmium can alter DNA methylation.

A first known mechanism is the effect on DNA methylation mediated by depletion of the methyl donor SAM, which in turn follows from either direct interaction of cadmium with GSH, or from GSH depletion through induction of ROS. SAM is also the methyl donor in DNA methylation, which may thus be decreased by cadmium through the depletion of SAM (Figure 7, KER10). Compensatory GSH production after its interaction with cadmium or its depletion after induction of ROS competes with SAM production because they both use the same precursor, homocysteine (Figure 7, KER9). Experimental evidence for a role of oxidative stress in DNA methylation comes from exposure with H_2_O_2_ in multiple cell lines, which led to the hypomethylation of long interspaced nuclear element-1 (LINE-1), a commonly used marker for genome wide methylation [40]; this DNA hypomethylation was associated with decreased SAM. Antioxidants (α-tocopheryl acetate and *N*-acetylcysteine) and supplementation of methyl donors methionine, SAM and folic acid restored LINE-1 methylation and reduced oxidative stress. However, whether these effects can be extrapolated to the in vivo situation is unclear.

On the other hand, cadmium is known to cause GSH depletion in multiple vertebrate species [9], and SAM depletion and subsequent DNA hypomethylation, although not specifically analyzed, can be assumed as a plausible result. The rescue in the above in vitro study increases the weight of the presented evidence, as it increases confidence for essentiality considerations. However, empirical evidence is low as the subject has been studied minimally, and it should be noted that the pathway through SAM depletion only explains how cadmium exposure can lead to DNA hypomethylation, but not to the also observed hypermethylation.

A further consideration is that cadmium toxicity may depend on the antioxidative potential. Increased susceptibility to cadmium was observed in rats where GSH depletion increased cadmium-induced 8-OHdG formation [41]. The redox potential during zebrafish development rapidly decreases during early stages (until 36 hpf), after which it rises to a steady state at 72 hpf and later [42]. This suggests that the susceptibility for the toxic potential of cadmium increases during periods of greatest cellular differentiation, and this critical period for cadmium toxicity thus coincides with susceptibility to altered programming.

Cadmium has a high affinity for thiol groups, including those present in GSH [43]. Excessive cadmium thus results in GSH inactivation [43], possibly among other antioxidants (Figure 7, KER7) [16,44]. This mechanism has been well studied, creating high biological plausibility. Also, cadmium-thiol binding is essential for GSH depletion, and GSH binding, being a first response, creates high empirical support for the KER. The same GSH inactivation through cadmium-thiol binding leads to accumulation of ROS, which contributes to the depletion of GSH (Figure 7, KERs 7, 8 and 11). Other affected molecules include the cysteine-rich metallothionein proteins, which also show antioxidative functions [43]. When antioxidative mechanisms are overrun, endogenous ROS will accumulate, producing oxidative stress.

Cadmium may also affect the oxidative stress defense system through inactivation of antioxidative enzymes (Figure 7, KER12), but reported results of acute cadmium exposure on the antioxidative enzymes catalase (CAT) and superoxide dismutase (SOD) are conflicting, showing variable up, down or no effects in various in vitro and in vivo models [45,46], including the above mentioned study in zebrafish embryos [16]. The CAT and SOD activity changes appeared to relate to the direct binding of cadmium via electrostatic forces, leading to enzyme misfolding in vitro (Figure 7, KER14), and the subsequent induction of oxidative stress in vivo [47]. However, this was not investigated in other studies, which leads to low confidence for this mechanism. Effects on antioxidative enzymes might also arise from the blockage of the cysteine residue in the active site after cadmium interaction with cellular thiols (Figure 7, KER15) [48]. However, the confidence for this claim is weak because there is no experimental evidence.

A negative effect on GPx activity can also come from depletion of selenium after its interaction with cadmium, which is a well characterized mechanism (Figure 7, KER14). Cadmium excretion is facilitated after binding to selenium [49]. Since selenium is an integral component of the catalytic site of GPx (Available online: uniprot.org/uniprot/P18283), a decrease in cellular selenium negatively affects GPx activity, resulting in a decreased oxidative stress defense [50].

Cadmium can also interfere with antioxidative enzymes through substitution of endogenous metals in the active site of metalloproteins (Figure 7, KER16), including SOD, CAT and other antioxidative enzymes. However, this is not a highly plausible factor in oxidative stress production, in view of the absence of robust empirical evidence and also because this mechanism probably depends on relatively high cadmium concentrations.

Finally, cadmium could induce oxidative stress through the indirect production of ROS, particularly via the induction of excessive free copper and iron ions after substitution in metalloproteins (Figure 7, KER17). These free ions can induce ROS via Fenton reactions (Figure 7, KER18). However, this is not a highly plausible major contributor to oxidative stress, because endogenous metal substitution by cadmium is questionable at low concentrations.

Still, all these oxidative defense inactivation mechanisms may, in combination, potentially contribute to less efficient degradation of endogenous ROS, leading to the accumulation of ROS and oxidative stress.

### 3.5. Methylome Alteration via Oxidative DNA Damage

One way cadmium-induced oxidative stress may lead to DNA methylation alteration, as reviewed by Franco et al. [51], is through the oxidation of bases in the CpG sequence (Figure 7, KERs 19 and 20). Primary products of ROS induced oxidative reactions with guanine in the DNA are 8-hydroxyl-2′-deoxyguanosine (8-OHdG) and 6-*O*-methylguanine, leading to a major decrease of DNMT binding to DNA, and thereby to its ability to methylate the neighboring cytosine residue in a CpG site [52,53]. Cadmium-induced generation of 8-OHdG has been observed in cultured cells in association with decreased GSH [54], in GSH-depleted rats [41], and in NiCad battery workers [55]. For this reason, we measured 8-OHdG in our cadmium-exposed zebrafish embryos, which suggested a dose-dependent increase of DNA oxidation up to the highest subtoxic concentration (Figure 2). The lower 8-OHdG at the highest toxic concentration compared to subtoxic concentrations may be due to other superimposed mechanisms. This mechanism is fairly well studied, providing moderate biological plausibility. However, it again would only produce hypomethylation, decreasing the confidence in empirical considerations.

Another way DNA oxidation may alter DNA methylation is through improper recruitment of de novo DNMTs. ROS can induce single strand DNA breaks (Figure 7, KER21), which signals de novo methylation [56]. This may thus represent another pathway through which cadmium-induced oxidative stress reshapes the methylome (Figure 7, KER22). Although this pathway has been studied minimally, it may, in contrast to other pathways described above, potentially explain cadmium-induced DNA hypermethylation, implicating larger confidence in empirical considerations.

Altogether, cadmium-induced oxidative stress may well explain the effect on DNA methylation.

Cadmium is shown to affect DNMT activity, as reviewed by Filipič [57]. Acute cadmium exposure inhibited overall DNMT activity in vitro and ex vivo [58] and in vivo [59]. The effect of cadmium on DNMT activity can be explained through competition with the zinc ion in the DNA binding zinc finger domain of DNMT (Figure 7, KER24), thus decreasing DNA interaction activity [60]. This decrease would lead to impaired enzyme function, and thus to hypomethylated DNA. Disruption of zinc finger based DNA binding by cadmium has been observed in other proteins [61,62], increasing biological plausibility, but empirical considerations (only high cadmium concentrations are effective for competition with zinc) decrease the likelihood of this competition with endogenous metals in metalloproteins being the major affected pathway leading to the observed apical phenotypes.

Alternatively, cadmium can inhibit DNMTs through binding to their cysteine residue [60] (Figure 7, KER25), thereby inhibiting the catalyzing function. However, there is no experimental evidence for this claim, and the plausibility of this being a major mechanism for cadmium induced DNMT inhibition is questionable, again due to the large number of other thiol-containing molecules indicating unspecific effects. This leads to low confidence for this mechanism.

In conclusion, though several molecular mechanisms are proposed for direct DNMT inhibition by cadmium, the plausibility of these mechanisms is questionable, due to the lack of DNMT specificity of the interactions.

### 3.6. Informative Key Events (KEs)

DNA methylation is a well-studied and understood KE and central in all described pathways, and is therefore probably optimal to predict long term apical phenotypes. DNA methylation is established early in life and persists to determine functions later in life. Methylome alteration studies should thus be predictive for delayed apical phenotypes, but this requires identification of specific DNA methylation markers which, in the zebrafish embryo, may be hampered by loss of tissue specific effects in the noise of the whole organism. Another informative candidate KE is DNA base oxidation, specifically 8-OHdG formation, which may precede DNA methylation alteration. Suggested effects at subtoxic concentrations in our model support further exploration of this endpoint. Other KEs, particularly in the antioxidative physiology pathway, are not specifically predictive for methylation alterations and therefore not suitable as markers for programming effects.

## 4. Materials and Methods

### 4.1. General Zebrafish Maintenance and Embryo Exposures

**Figure 8:**
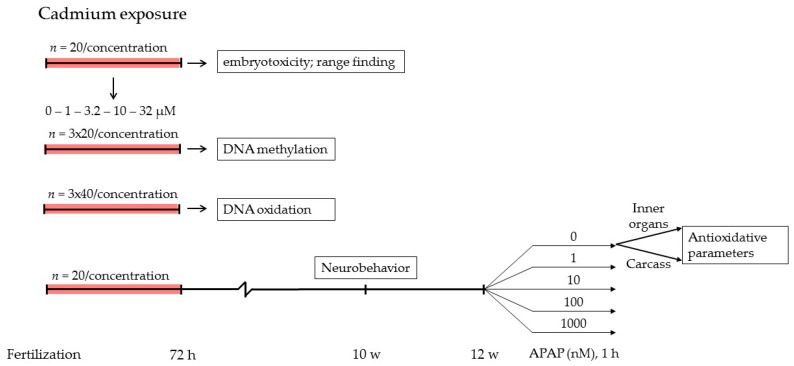
Workflow for the experiments in this study. Cadmium exposure was from 0–72 hpf in all experiments. Neurobehavioral analysis was started at 10 and antioxidative analysis at 12 weeks (w) post fertilization.

Zebrafish maintenance and embryonal exposures were as reported previously [7]. In short, zebrafish were housed in a ZebTec flow-through system with 7.5 L tanks, a light-dark cycle of 14–10 h, and automatically controlled water conditions, including a temperature of 26 ± 1 °C, pH at 7.5 ± 0.5, conductivity at 500 ± 100 µs/cm, and CaO levels at 10–250 mg/L. Adult fish received powdered feed (Special Diet Services, Essex, UK) twice a day and frozen *Artemia* (Ruto, Montfoort, The Netherlands) once a day. Juvenile feeding consisted of *Brachionus calyciflorus* for 5–11 days post fertilization (dpf), freshly hatched live *Artemia* for 12–18 dpf and powdered feed and live *Artemia* from 19 dpf onwards. First, range-finding experiments with cadmium were done with individually exposed embryos (one embryo per well in a 24-wells plate (Greiner Bio-One, Alphen aan den Rijn, The Netherlands) with 2 mL medium) from 0–72 h post fertilization (hpf) to determine embryotoxic effects, using a scoring system for an extended set of developmental and teratological hallmarks (adapted OECD test guideline 236 [63]); see Figure 8 for the work flow. For analysis of effects on DNA methylation in the embryos, pools of embryos were exposed from 0–72 hpf in petri-dishes (20 embryos per dish with 2 mL medium per embryo). Medium renewal was not applied in this nor in the following exposure protocols. A further experiment was then initiated to analyze long term effects of embryonal exposure. For the latter, freshly spawned, fertilized zebrafish eggs were individually exposed from 0–72 hpf to a blank control or a nominal CdCl_2_ concentration determined in range-finding experiments (1.0, 3.2, 10 and 32 µM), 20 embryos per concentration, each in a volume of 2 mL in 24-wells plates (Greiner Bio-One, Alphen aan den Rijn, The Netherlands). After exposure, zebrafish were transferred to control water (1.2 mM NaHCO_3_, 0.20 mM KHCO_3_, 1.4 mM CaCl_2_ and 0.73 mM MgSO_4_, saturated O_2_) and housed per exposure group of 20 in glass 1 L tanks placed in a temperature-controlled water bath. Water was constantly aerated and refreshed twice a week. At approximately 10 and 12 weeks of age, the zebrafish were subjected to neurobehavior analysis and antioxidative parameter measurement, respectively. Feeding was terminated at 24 h prior to antioxidative parameter measurement to minimalize interference. The study was approved by the local animal experiments ethical committee (DEC RIVM 201400212).

### 4.2. DNA Methylation

Effects on DNA methylation were analyzed in 72 hpf whole embryo DNA extracts (each extract containing one pool of 20 embryos) in promoter regions of three genes *cyp19a2*, *vasa*, and *vtg1*, using a pyrosequencing method. This analysis method was described previously [7].

### 4.3. DNA Oxidation

DNA oxidation was measured after isolation of DNA from triplicate pools of 40 embryos in the same concentration range as before under oxidation protecting conditions by the NaI method using the DNA Extractor WB Kit 291-50502 (Wako Chemicals USA, Inc., Richmond, VA, USA) with the protocol following the DNA extractor TIS kit 296-67701 (Wako). Briefly, pools of about 40 zebrafish embryos were collected in a microtube and homogenized with a pestle in ice-cold lysis solution containing a non-ionic surfactant, polyoxyethylene oxyphenyl ether, centrifuged at 600× *g* for 10 min at 4 °C. The pellet was washed twice in ice-cold lysis solution, resuspended in 200 µL enzyme reaction solution with 2.5 µL RNase cocktail (Ambion, Austin, TX, USA), mixed by vortexing vigorously and incubated at 37 °C for 10 min. Then, 10 µL protease solution was added and incubated for a further 30 min. Samples were then centrifuged at 10,000× *g* for 5 min at room temperature. The supernatant was collected and mixed with NaI solution, DNA was precipitated, washed and dissolved according to the manufacturer′s protocol and stored at −80 °C. DNA concentrations were measured by NanoDrop spectrophotometer (Isogen Life Science, De Meern, The Netherlands). An amount of 25 µg of DNA was digested to mononucleotides with nuclease P1 using the 8-OHdG Assay Preparation Reagent Set (Wako) according to the manufacturer′s protocol. The final hydrolysates were filtered by centrifugation through VIVASPIN 500 30,000 MW cutoff (Satorius, Goettingen, Germany).

The level of 8-OHdG was measured using the standards 8-hydroxy-2-deoxyguanosine, 2-deoxycytidine, 2-deoxyguanosine monohydrate (all Sigma, St. Louis, MO, USA), and 5-methyl-2-deoxycytidine (MP Biomedicals LLC, Santa Ana, CA, USA) by chromatographic separation using a Shimadzu Nexerra ultra high performance liquid chromatography (UHPLC) system fitted with an high strength silica C18 column (150 mm 2.1 mm i.d., 1.8 µm, Waters Chromatography, Milford, MA, USA placed in a column oven at 40 °C, with an operating flow of 0.25 mL/min. The gradient started with 10% mobile phase B (acetonitrile acidified with formic acid) and 90% mobile phase A (ultra liquid chromatography/mass spectrometry (ULC/MS) gradewater acidified with 0.1% formic acid), after 5 min the gradient increased in 1 min to 90% mobile phase B and held for 2 min. Finally, the gradient was changed to 10% mobile phase B and held for 2 min; solvents used for the chromatographic separation were ULC/MS grade (Biosolve B.V., Valkenswaard, The Netherlands). The injection volume was 50 µL. Detection of the analytes was performed using a Sciex Qtrap 6500 mass-spectrometer with electrospray ionization source operating in the positive mode. The operating parameters were, Curtain gas: 30, Collision Gas: Medium, Ionspray voltage: 5500, Ion Source Temperature: 400, Ionsource Gas 1:20, Ionsource gas 2:0. The components were fragmented by N_2_ dissociation. See Supplementary Table S1 for the settings of the Selected Reaction Monitoring transitions. Data was collected with the Analyst software version 1.6.2 and processed using MultiQuant software version 3.0.1 The results are expressed as the ratio of nmol of 8-OHdG to 10^5^ nmol of 2-deoxyguanosine.

### 4.4. Neurobehavior

Neurobehavior of a 10 week old, embryonically exposed zebrafish was analyzed using the Viewpoint video tracking system (Viewpoint, Lyon, France), which enables detection in horizontal and vertical planes, as well as movement analysis in dark environments, using an infrared source and camera. Videos were analyzed using Viewpoint software to determine spatial and temporal movement parameters, where the average output over a 10 min period was determined as sufficiently informative in pilot measurements with control fish. The analyses included basal movement and measurements in different environments or after the induction of stress.

*Novel tank diving.* To induce stress, zebrafish were transferred from their home tanks into a novel environment, i.e., a plastic 1 L tank filled with 800 mL ZebTec system water, using a net. The natural response of the fish is to stay at the bottom (bottom dwelling) for a few minutes until the stress of the transfer wore off [64]. Time spent at the bottom third of the tank per 60 s was reported.

*Tapping response.* To assess response to a stressor, the tapping test was performed [65]. After a baseline measurement, a tapping mechanism (custom engineered by Jidee, Amstelveen, The Netherlands) tapped a small metal pin against the plastic tank at a frequency of one tap per 10 s for 3 min. Zebrafish responded by short, fast movements, i.e., “burst” movements, defined as >100 mm/s. The burst distance (distance travelled at >100 mm/s) was measured and, from this, the burst distance ratio was calculated, i.e., average control burst distance to the average first minute tapping burst distance.

*Dark/light preference.* Zebrafish prefer light over dark environments [64,66], and therefore dark/light preference can be used as another parameter of normal behavior. Half of the bottom and sides of the transparent tank was covered with black paper to create light and dark areas in the left and right half of the tank, respectively, with measurement of the relative time spent in the light compartment.

*Color preference.* To assess sensory behavior, the color preference was performed with bottom and sides of each left and right half of the tank covered with blue and orange paper. Zebrafish have a preference for orange over blue [67]. Time spent in the orange compartment was measured.

Testing was performed with individual zebrafish over the course of several days, with balanced distribution of samples per concentration over the days and time of day. The testing sequence started with the novel tank diving test, followed by the remainder of the tests on the next day. Each test was preceded by a period of 5 min for acclimatization and recovery of tank handling. Experiments were performed at a controlled room temperature of 26 ± 1 °C with a photoperiod of 14-h light:10-h dark.

### 4.5. Oxidative Stress Defense

For oxidative stress evaluation, multiple antioxidative parameters were measured in zebrafish internal organs and the remaining carcass. This was done to differentiate effects between organs with relative high (internal organs) or low (carcass) values of the measured parameters. Reduced and oxidized glutathione (GSH and GSSG) concentrations, glutathione peroxidase (GPx) activity, glutathione reductase (GR) activity, superoxide dismutase (SOD) activity, total thiol (TTT) concentration and the biological antioxidant potential (BAP) were measured.

Reduced glutathione (GSH) is the main antioxidant in many species, including humans and zebrafish. The main function is to prevent cell damage from reactive oxygen species (ROS). ROS capture by GSH is catalyzed by glutathione peroxidase (GPx) which oxidizes two GSH molecules, forming the oxidized glutathione dimer (GSSG). GSSG can be recycled to GSH by glutathione reductase (GR).

Superoxide dismutase (SOD) is a ubiquitous enzyme which converts superoxide to hydrogen peroxide, which is easier to remove, and oxygen (${{2O}_{2}}^{-}+{2H}^{+}\to H_{2}O_{2}+O_{2}$). This is a first line of defense against ROS in most species.

The biological antioxidant potential and total thiols parameters are used more as an indication for the antioxidative physiology response, since a range of compounds and no specific biological function is measured. The biological antioxidant potential indicates the ability to eliminate ROS through antioxidants. A wide range of antioxidants is present in zebrafish, both endogenous and exogenous. Change in the biological antioxidant potential suggests a modified defense against oxidative stress. Thiols are compounds that contain a sulfhydryl group (–SH) attached to a carbon atom. Thiol containing molecules assist a cell in maintaining a reducing state by binding oxidizing agents. Total thiol (TTT) measurement relates strongly to GSH measurement, but takes additional molecules into account.

To induce oxidative stress, zebrafish were challenged with 1, 10, 100 or 1000 nM acetaminophen (APAP, paracetamol) in water for one hour. Next, zebrafish were euthanized using ice water and kept herein until dissection. Internal organs, including intestines, liver/pancreas, testis/ovary, kidneys, spleen, and swim bladder, were removed *en bloc* and both the internal organs and the remaining carcass were flash frozen in liquid nitrogen and stored overnight at −80 °C. The following day samples were homogenized in 0.1 M Tris-HCl (pH 7.4). Samples were then measured using the Beckman Coulter UniCel DxC800, a pipetting robot with a spectrophotometer.

Glutathione reagents were commercially purchased from Cayman Chemical company, United States (Cat. No. 703002); GR, GPx and SOD reagents from Randox Laboratories Limited, United Kingdom (Cat. No. resp. GR 2368, RS 504/5 and SD 125); BAP reagents from DIACRON Labs S.r.l., Italy (Cat. No. MC436/7) and TTT reagents from Rel Assay Diagnostics, Turkey. Assays were performed per manufacturer′s instructions.

### 4.6. Statistical Analysis

All experiments were designed in a dose-response set-up to enable benchmark analysis with PROAST software [68]. PROAST applies fitting an increasingly complex mathematical model to concentration response data and calculating a specific concentration (the Critical Effect Dose, CED; or Benchmark Dose, BMD) to a pre-defined response (the Critical Effect Size; CES, or Benchmark Response; BMR). The most optimal model is defined by statistical significant departure from the no-effect model (*y* = *a*). When covariates are used in the analysis (as applied for cadmium concentrations in the time-response analysis, Figure 4; and in the APAP concentration analysis, Figure 6), the covariate parameters produce separate responses if their differences are statistically significant. Conclusions on statistical significance are implicit to the output of models that differ from *y* = *a* or when different curves are given for covariate conditions. The ratio of the upper and lower bound of the confidence interval at CED (CEDU/CEDL) was used as a measure of precision of the result, and considered acceptable at approximately 10 or less. All calculated exponential models were confirmed by a Hill model analysis with the same parameters. In our analyses, PROAST was used to detect statistically significant dose responses, and CEDs were of no further use.

### 4.7. Hypothesized Adverse Outcome Pathway (AOP) Framework Development

The AOP network construction was based on the hypothesized pathway described in the Introduction. A middle-out approach was used, as alteration in the methylome was already a proposed KE. Thus, we first explored the plausibility of pathways from a changed methylome to the observed adverse outcomes (AOs, neurobehavior and antioxidative physiology). Next, molecular initiation events (MIEs) were identified and pathways leading to a changed DNA methylome were investigated. This middle-out approach produced multiple up- and downstream pathways, MIEs and AOs. The elements of the hypothesized AOP framework were derived from (recent) reviews and epidemiological and experimental studies describing molecular cadmium toxicity and/or epigenetics—the latter selected to be similar to our experimental setup.

Weight of evidence was assessed based on tailored Bradford-Hill considerations for KERs [69], including biological plausibility, essentiality and empirical evidence. Biological plausibility was assessed by questioning whether there is established biological knowledge consistent with the KER. Understanding of the mechanistic relationship between the two KEs increases robustness of the pathway. Next, evidence for essentiality is considered. This describes the importance of a KE by determining whether the downstream KEs (and AO) can be prevented when an upstream KE is blocked. Finally, empirical evidence is reviewed, including parameters such as concordance, timeframe and dose-response relationships. For the KERs, we focused only on considerations of major relevance (positive and negative).

## 5. Conclusions

Embryonic cadmium exposure has effects on zebrafish neurobehavior later in life, as concluded from the measurement of baseline movement and the novel tank diving test. Embryonic cadmium exposure also produced a dose-response effect later in life on antioxidative physiology and affected glutathione peroxidase effectiveness when oxidative stress was induced. The observed effects in both neurobehavioral and antioxidative physiology can be explained by either programming effects or by direct developmental toxicity. Altered programming is the most plausible pathway since apical phenotypes were observed at concentrations far below observed direct toxicity, and it explains observed effects in these unrelated functions in the organism. Furthermore, altered neurobehavior and antioxidative physiology have been explained by altered programming in other models.

Although no reproducible effects were observed in the methylation of three specific DNA targets, DNA methylation was proposed as the foremost epigenetic mechanism for altered programming because it is highly involved in cell differentiation and thus shaping the phenotype during early development, which is the time frame used in our exposure study.

Depletion of SAM, oxidative changes to DNA, and direct DNMT inhibition were proposed for cadmium affecting DNA methylation. Depletion of SAM through increased GSH biosynthesis due to direct cadmium interaction with GSH or to ROS accumulation is plausible as it is fairly specific for cadmium and other heavy metals, but the mechanism only explains DNA hypomethylation. Cadmium induced methylome alterations may thus be inflicted via multiple pathways with a common MIE. There is support from literature for the generation of 8-OHdG after cadmium exposure, although the observation in our model is inconclusive. Direct inhibition of DNMTs by cadmium seems improbable due to the lack of specificity for cadmium and DNMTs.

Altogether, our study confirms the late life effects of embryonic cadmium in zebrafish and proposes a pathway that describes embryonic cadmium leading to long term apical phenotypes. The challenge remains to identify specific and informative targets of altered DNA methylation.

## Figures and Tables

**Table 1:** Effects of cadmium on DNA methylation in 72 h post fertilization (hpf) zebrafish embryos.

Target	*cyp19a2*-1	*vasa*-3	*vasa*-5	*vtg1*-3
effect	-/↑ 164	↑ 61.6/-	↑ 143/-	-/↑ 74.7

The fragments of the three targets contained seven (*cyp19a2*) or three (*vasa*, *vtg1*) analyzable CpGs; the affected CpG is given after the hyphen. Effects are provided as CED05 (µM) in two respective experiments, separated with a slash (↑, hypermethylation; -, no effect).

**Table 2:** Effects of embryonic CdCl_2_ exposure on baseline values of antioxidative parameters.

Parameter	Carcass (μM)	Organ (μM)
GPx	0.46 (0.20–1.3)	0.30 (0.04–2.8)
GSH	0.01 (0–0.04)	0.04 (0–0.42)
GSSG	0.15 (0–1.3)	0.47 (0.17–0.92)
GSSG:GSH ratio	0.01 (0–0.11)	0.01 (0–0.54)
TTT	14.1 (9.16–31.09)	0.10 (0.04–0.27)
GR	ns	ns
SOD	ns	ns
BAP	ns	ns

Internal organs were removed from the body and extracted separately from the remainder of the carcass for measurement of antioxidative parameters. Values are 5% critical effect doses (CED05) with upper and lower 95% confidence interval limits (CEDL, CEDU) in brackets (ns: not significant).

## Supplementary Materials

Supplementary materials can be found at www.mdpi.com/1422-0067/17/11/1830/s1.

## Abbreviations

NCD	Non-communicable disease
DOHaD	Developmental origins of health and disease
SAM	*S*-adenosyl-l-methionine
DNMT	DNA methyl transferase
AOP	Adverse outcome pathway
MIE	Molecular initiating event
KE	Key event
KER	Key event relationship
dpf	Days post fertilization
GSH	Reduced glutathione
GSSG	Oxidized glutathione
GPx	Glutathione peroxidase
GR	Glutathione reductase
SOD	Superoxide dismutase
TTT	Total thiols
BAP	Biological antioxidant potential
ROS	Reactive oxygen species
APAP	Acetaminophen, paracetamol
CED	Critical effect dose
8-OHdG	8-Hydroxyl-2′-deoxyguanosine
