# Supplementary material for: Programmed Effects in Neurobehavior and Antioxidative Physiology in Zebrafish Embryonically Exposed to Cadmium: Observations and Hypothesized Adverse Outcome Pathway Framework
Source: Int J Mol Sci. 2016 Nov 2;17(11):1830. doi: 10.3390/ijms17111830 (PMC5133831; doi:10.3390/ijms17111830)
Supplement: Supplementary file 1 [file ijms-17-01830-s001.pdf]

Supplementary Material: Programmed Effects in Neurobehavior and Antioxidative Physiology in Zebrafish Embryonically Exposed to Cadmium: Observations and Hypothesized Adverse Outcome Pathway Framework

Sander Ruiter, Josefine Sippel, Manon C. Bouwmeester, Tobias Lommelaars, Piet Beekhof, Hennie M. Hodemaekers, Frank Bakker, Evert-Jan van den Brandhof, Jeroen L. A. Pennings and Leo T. M. van der Ven

Table S1. Selected reaction monitoring transition parameters.

Compound	Q1	Q3	Time (msec)	Declustering Potential	Entrance Potential	Collision Energy (volts)	CXP (volts)
8-OHdG	284.1	168.0	100	11	10	17	10.0
8-OHdG	284.1	140.0	100	11	10	43	14.0
2-dC	228.0	112.0	100	20	15	20	10.5
2-dC	228.0	95.0	100	20	5	50	10.5
2-dG	268.1	152.1	150	71	10	15	10.0
2-dG	268.1	134.9	150	71	10	49	14.0

Q1, Q3, quadrupole 1, 3; CXP, Collision cell exit potential; 8-OHdG, 8-hydroxy-2-deoxyguanosine; 2-dC, 2-deoxycytidine; 2-dG, 2-deoxyguanosine.
